# Prevalence and Predictors of Food Insecurity Among Medical Students During Clinical Training: A Cross-Sectional Study

**DOI:** 10.7759/cureus.97685

**Published:** 2025-11-24

**Authors:** Reem Saab, Ozdemir Erdemir, Lumnie Lika, Megan Jackson, Yue Yin, Alexandra Johnston, Mary Lynn Sealey

**Affiliations:** 1 Department of Obstetrics and Gynecology, Allegheny Health Network, Pittsburgh, USA; 2 Department of Internal Medicine, Temple University Hospital, Philadelphia, USA; 3 Department of Medicine, Drexel University College of Medicine, Philadelphia, USA; 4 Department of Internal Medicine, Allegheny Health Network, Pittsburgh, USA; 5 Allegheny Singer Research Institute, Allegheny Health Network, Pittsburgh, USA

**Keywords:** food insecurity, graduate medical education, medical students, nutrition, social determinants of health

## Abstract

Introduction: Food insecurity is a growing concern in higher education and may adversely affect medical students’ academic performance, well-being, and professional development. Although awareness of this issue is increasing in graduate populations, limited data exist regarding its prevalence and associated factors within medical education. This study aimed to assess the prevalence of food insecurity among medical students completing clinical rotations and to identify demographic and institutional factors associated with increased risk.

Methods: We conducted a cross-sectional study of third- and fourth-year medical students from two US medical schools who were completing clinical rotations at a large academic health system during the 2022-2023 academic year. All 75 eligible students were invited to participate, and all completed an anonymous online survey distributed through Qualtrics. Food insecurity was assessed using the 10-item United States Department of Agriculture Adult Food Security Survey Module, and scores were categorized according to standard food security levels. Demographic variables collected included age, race/ethnicity, academic year, medical school, financial aid status, primary residence, and use of food-related resources. Descriptive statistics characterized participants, and group differences were analyzed using t-tests, ANOVA, and univariate and multivariate linear regression to identify predictors of food insecurity.

Results: All 75 students responded (100% response rate). The median age was 26 years, and most identified as White (47 or 62.7%) or Asian (17 or 22.7%). The overall mean food insecurity score was 2.11 - more than three times the US national average. Students from Institution A had significantly higher scores than those from Institution B (2.71 vs. 0.53; p < 0.0001). Students receiving financial aid also reported higher scores than those not receiving aid (2.33 vs. 0.80; p = 0.0137). In multivariate regression, only the medical school attended remained a significant predictor (p = 0.0125), with Institution A students scoring 1.87 points higher on average. No significant associations were observed for academic year, race/ethnicity, or resource use.

Conclusion: Food insecurity appears to be more prevalent among medical students than in the general population, underscoring an important yet underrecognized challenge during clinical training. Institutional differences in food insecurity suggest that campus-specific or environmental factors may contribute to disparities in access, while the lack of significance for financial aid in adjusted analyses indicates the influence of additional structural barriers. These findings highlight the need for targeted, institution-specific support strategies and further research to develop effective interventions that improve food access and promote medical student well-being.

## Introduction

Food insecurity is highly prevalent among both undergraduate and graduate students in the United States, with rates consistently exceeding those of the general population [1,2]. Multiple studies and systematic reviews report prevalence estimates ranging from 15% to over 40%, depending on the institution, region, and methodology used [3-8]. For example, a large multi-institutional study found that 19% of first-year college students were food insecure, with an additional 25% at risk [9]. Another national analysis reported a 40% prevalence among postsecondary students [5].

There is a strong association between food insecurity and poor physical health, mental health challenges, and reduced academic performance among students [1,3,10]. Additionally, malnutrition, chronic stress, and fatigue - factors often linked with food insecurity - may collectively hinder students’ ability to excel in their studies and clinical responsibilities [10-12]. While previous studies have extensively explored the prevalence of food insecurity among graduate students [1,3,11,13], it is imperative to examine how these challenges manifest within the unique context of medical education.

Graduate and medical students face a myriad of challenges, including long and unpredictable hours, constrained time and resources, and heightened stress levels, all of which can contribute to an environment conducive to food insecurity. Although direct quantitative evidence linking long hours or stress specifically to food insecurity among medical students is limited, several qualitative studies provide relevant associative data that strengthen this argument. A qualitative study of Harvard medical students found that food-insecure participants described time constraints and academic pressures as key barriers to obtaining affordable, nutritious food, noting that their demanding schedules left little time for meal preparation or shopping [14]. Students also reported that food insecurity itself increased stress, creating a negative feedback loop that affected well-being and academic performance [14]. Similarly, a qualitative study at a diverse urban university identified insufficient time to prepare and eat meals, as well as stress and anxiety about food access, as central experiences among students with marginal food security. While large-scale quantitative studies in medical students have focused primarily on sociodemographic and financial predictors of food insecurity [15], the qualitative evidence above demonstrates that long hours and heightened stress are perceived as significant contributors. Additionally, studies in broader college populations have shown that food insecurity is associated with higher stress and poorer mental health, suggesting a bidirectional relationship between stress and food insecurity [16-18]. Taken together, these findings suggest that the demanding schedules and psychological stress inherent in medical education may both precipitate and be exacerbated by food insecurity, reinforcing the need for targeted investigation in this population.

Although food insecurity affects a wide range of populations, understanding its implications within medical education is critical given the demanding nature of training and its potential influence on academic performance, well-being, and future professional development. Therefore, this study aimed to (1) assess the prevalence of food insecurity among medical students completing clinical rotations and (2) identify demographic and institutional factors associated with increased risk. By focusing on students during their clinical years - when long hours and stress are most pronounced - this study seeks to provide actionable insight into the scope and predictors of food insecurity within medical education.

## Materials and methods

Study design and population

This cross-sectional study assessed the prevalence of food insecurity among medical students by surveying students from two US medical schools (Institution A and Institution B), who were completing their clinical rotations at a large academic health system. We included all third- and fourth-year students who were rotating at this health system during the 2022-2023 academic year. No students were excluded, and the survey was distributed to a total of 75 students.

Survey design and distribution

We assessed survey participants for food insecurity and collected demographic information, including age, race/ethnicity, academic year, medical school, and use of food-related resources. All questions in the demographic portion of the survey are shown in Appendix A.

Food insecurity status was determined using the United States Department of Agriculture (USDA) 10-item Adult Food Security Survey Module [19]. This survey tool is widely used in public health and nutrition research and has demonstrated strong reliability and validity across diverse populations. It is considered a gold-standard instrument for assessing food insecurity in the United States and has been validated in college and graduate student populations [20]. The survey included questions regarding multiple aspects of food security, including hunger, food sufficiency, reduced food intake, anxiety about food, skipping meals, inadequate nutrition, reduced food variety, and overall diet quality. Food security levels were categorized using the USDA scoring system, and each participant’s total score determined their level of food security. Scores ranged from 0 to 10 and were classified as high (0), marginal (1-2), low (3-5), and very low (6-10) food security.

The survey was distributed electronically via Qualtrics, a secure online platform, between February 1 and April 30, 2023. It was sent five times via email reminders. The survey system assigned a unique identifier to each participant to ensure anonymity while preventing duplicate submissions. The food insecurity assessment referenced participants’ experiences during the preceding three months.

Rationale for Tool Selection: The 10-item USDA Adult Food Security Survey Module was selected because it allows for comparability with national data and with prior studies on food insecurity in higher education, facilitating benchmarking across populations [1-3,9,11]. Its brevity and validated scoring make it well-suited for busy medical student respondents, minimizing survey fatigue while maintaining psychometric reliability. Although other versions, such as the 18-item household survey or abbreviated six-item modules, exist, the 10-item format strikes an optimal balance between depth and response burden. Prior studies have successfully adapted and validated this tool for use in graduate and health science trainee populations [12,20], supporting its appropriateness for the present study.

Statistical analysis

Descriptive statistics were calculated for demographic variables to characterize the study population. ANOVA was used to compare food insecurity scores among ethnic groups. Differences between the two groups (e.g., financial aid status) were assessed using independent t-tests. Univariate and multivariate linear regression analyses were performed to assess predictors of food insecurity scores, including academic year, medical school, resource use, and financial aid. Predictors with a p-value < 0.25 in univariate analysis were included in the multivariate model. All statistical analyses were performed using SAS 9.4 with an alpha level of 0.05 for significance.

## Results

Our study achieved a 100% participation rate, as all 75 eligible students completed the survey. The median age of the study population was 26 years (IQR: 25-28). Most participants identified as White (47, 62.7%), followed by Asian (17, 22.7%), Black (3, 4.0%), and Hispanic/Latino (3, 4.0%), with five students (6.7%) identifying as “Other” (Table 1). The majority of respondents were third-year students (53, 69.7%) enrolled at Institution A (56, 73.7%) or Institution B (20, 26.3%).

**Table 1:** Demographic Characteristics of Medical Students Rotating at Academic Health Network (Institution A and Institution B, 2022–2023 Academic Year) IQR: interquartile range

Variables	Median (IQR)
Age (years)	26 (25-28)
Race/Ethnicity	n (%)
White	47 (62.67)
Black	3 (4.00)
Asian	17 (22.67)
Hispanic, Latino, or of Spanish origin	3 (4.00)
Other	5 (6.67)
Medical school year	
Year 3	53 (69.74)
Year 4	23 (30.26)
Medical school	
Institution A	56 (73.68)
Institution B	20 (26.32)

We compared food insecurity scores between students based on medical school, academic year, race/ethnicity, primary residence status, financial aid status, and resource use (Table 2). Mean food insecurity scores were significantly higher among Institution A students than Institution B students (Institution A mean = 2.71 vs. Institution B mean = 0.53; p < 0.0001). Students receiving financial aid also had significantly higher food insecurity scores (mean = 2.33) compared with those without financial aid (mean = 0.80; p = 0.0137). No statistically significant differences were found based on academic year, race/ethnicity, primary residence status, or resource use (Table 2).

**Table 2:** Food Insecurity Score Comparisons Among Third- and Fourth-Year Medical Students Rotating at the Academic Health Network, Based on USDA 10-Item Survey Score (Range: 0–10; Higher Scores Indicate Greater Food Insecurity) DF: degrees of freedom, SD: standard deviation. *Resources include SNAP, healthy food centers, and food pantries

Variables	Mean (SD)	DF	F value/T value	p-value
Race/Ethnicity		4	1.63	0.1765
Asian	1.13 (1.85)	-	-	-
Black	0.50 (0.71)	-	-	-
Hispanic	3.0 (3.0)	-	-	-
Other	4.40 (3.29)	-	-	-
White	2.20 (2.91)	-	-	-
Medical school year		68	1.37	0.1765
Year 3	2.42 (2.80)	-	-	-
Year 4	1.45 (2.58)	-	-	-
Medical school		63.84	4.8	< .0001
Institution A	2.71 (2.99)	-	-	-
Institution B	0.53 (0.77)	-	-	-
Family residence within 10 miles of clinical campus		68	0.06	0.9547
No	2.15 (2.27)	-	-	-
Yes	2.11 (2.87)	-	-	-
Resource use*		68	-1.2	0.2353
No	1.86 (2.86)	-	-	-
Yes	2.71 (2.45)	-	-	-
Financial aid recipient		24.23	-2.66	0.0137
No	0.80 (1.40)	-	-	-
Yes	2.33 (2.87)	-	-	-

Univariate and multivariate linear regression models were used to identify predictors of food insecurity scores (Table 3). Univariate analysis identified medical school as a significant predictor (p = 0.0026). This remained significant in the multivariate model (p = 0.0125). Institution A students had mean food insecurity scores 1.87 points higher than Institution B students (β = 1.87, p = 0.0125, R² = 0.16). Other variables, such as academic year, resource use, and financial aid, were not statistically significant predictors.

**Table 3:** Univariate and Multivariate Linear Regression Analysis of Predictors of Food Insecurity Scores Among Third- and Fourth-Year Medical Students (Score Range: 0–10, Higher Indicates Greater Insecurity) *Refers to students' in-state vs. out-of-state residency status as reported during clinical training **Resources include SNAP, healthy food centers, and food pantries

Variables	Univariate		Multivariate	
	Estimate	p-value	Estimate	p-value
Age	-0.1	0.331	-	-
Race/Ethnicity				
White	Reference	-	-	-
Asian	-1.07	0.193	-	-
Black	-1.7	0.3899	-	-
Hispanic	0.8	0.6261	-	-
Other	2.2	0.0924	-	-
Medical school year				
Year 3	Reference	-	-	-
Year 4	0.96	0.1765	0.56	0.4183
Medical school				
Institution B	Reference	-	-	-
Institution A	2.18	0.0026	1.87	0.0125
Primary residence*				
Yes	Reference	-	-	-
No	0.05	0.9547	-	-
Resource** use				
Yes	Reference	-	-	-
No	-0.86	0.2353	-0.37	0.5956
First-generation college student				
Yes	Reference	-	-	-
No	0.004	0.9955	-	-
First-generation medical student				
Yes	Reference	-	-	-
No	0.84	0.3111	-	-
Financial aid recipient				
Yes	Reference	-	-	-
No	-1.53	0.1034	-1.22	0.1783

## Discussion

Our study found that regional campus medical students reported food insecurity scores more than three times higher than the general US population [2]. Specifically, Rabbitt et al. [2] reported a national average score of 0.70, whereas our sample had a markedly higher mean of 2.11. This elevated prevalence highlights food insecurity as a critical and under-recognized issue within medical education.

Despite growing awareness of student food insecurity in higher education, several factors likely contribute to its limited recognition in medical schools. First, institutional culture in medical education often emphasizes resilience and self-sufficiency, which may discourage students from disclosing food-related hardship or seeking assistance. Second, because medical schools frequently affiliate with teaching hospitals rather than main university campuses, students may have reduced access to institutional food resources, such as pantries or subsidized meal programs. Third, food insecurity among medical students can be masked by socioeconomic assumptions - namely, that those pursuing professional degrees are financially stable - resulting in a lack of targeted screening or data collection within this population. Finally, the demanding structure of clinical training limits students’ time and flexibility to access food support services even when they exist. Collectively, these institutional and cultural barriers may delay recognition and hinder intervention efforts.

We also found that Institution A students reported higher food insecurity scores than Institution B students, suggesting that institutional and environmental factors may influence food access during clinical training. These findings align with prior work in undergraduate and graduate populations, which consistently highlight food insecurity as a widespread challenge in higher education [3,6,10,11]. While financial aid status was associated with higher food insecurity scores in bivariate analysis, the relationship was not statistically significant in the multivariate model. This suggests that financial aid alone may not fully mitigate food insecurity among medical students. Payne-Sturges et al. [9] similarly reported that financial aid does not adequately shield students from food insecurity, possibly because living costs and clinical demands exceed available support.

Although much of the literature on campus-based interventions focuses on undergraduates, some institutions have implemented programs - such as food pantries, meal subsidies, and financial literacy education - that could potentially support medical students as well [6,9]. However, given medical students’ older age, distinct schedules, and professional training demands, such interventions may require adaptation to be effective. Future research should explore the feasibility, uptake, and impact of food-support initiatives specifically within graduate medical education.

Our findings provide a foundation for larger, multi-institutional studies aimed at confirming patterns and informing targeted interventions to address food insecurity in medical students. Longitudinal research will be essential to measure intervention effectiveness - tracking changes in food-security scores, stress, and academic outcomes over time - and to determine whether institutional programs (e.g., meal stipends, flexible rotation meal access, or confidential screening) lead to sustained improvements. Mixed-methods or repeated-measure designs could capture both quantitative shifts and qualitative experiences of students as interventions are implemented. Qualitative studies could further elucidate the lived experiences, coping mechanisms, and resource barriers faced by food-insecure medical students.

Limitations

This study has several limitations. First, the cross-sectional design precludes causal inference and prevents assessment of how food insecurity may change over time during medical training. Second, no formal a priori sample size calculation was performed; thus, the study may have been underpowered to detect smaller differences among subgroups. Third, because the sample was limited to students from two medical schools rotating within a single health system, generalizability to other institutions may be limited. Fourth, we did not collect data on additional potential confounding variables, such as income, housing stability, or institutional food policies, which may have influenced food security status. Fifth, the use of a self-administered online survey distributed via Qualtrics may introduce response and selection bias, as participation was voluntary, and students experiencing food insecurity may have been more motivated to respond.

Despite these limitations, the study has notable strengths. It utilized a validated, nationally standardized assessment tool (the USDA 10-item Adult Food Security Survey Module) and achieved a 100% participation rate among eligible students. Furthermore, the use of multivariate regression analysis allowed the identification of independent predictors of food insecurity, strengthening the robustness of the findings. Taken together, this study provides valuable preliminary evidence on an underexamined issue in medical education and can serve as a foundation for larger, multi-institutional, and longitudinal studies designed to evaluate causal mechanisms and intervention effectiveness.

## Conclusions

Food insecurity appears to be more prevalent among medical students than in the general population, underscoring a critical but under-recognized concern during the clinical years of medical training. Institution A students reported significantly higher food insecurity scores than their Institution B peers, suggesting that institutional and environmental factors may influence disparities in food access. Beyond financial limitations, the findings point to structural contributors such as long hours, stress, and limited time for food preparation - factors intrinsic to medical training that are rarely captured in traditional quantitative assessments. The identification of these institutional differences highlights an opportunity for medical schools to develop tailored support programs that reflect the unique demands of clinical education, including flexible meal options, food-assistance referrals, and integration of well-being resources.

Future research should expand to multi-institutional and longitudinal designs to better quantify changes in food security status over time and to evaluate the effectiveness of interventions - such as meal stipends, campus food pantries, or confidential screening programs - on student well-being and academic performance. Qualitative studies should continue to explore the lived experiences of medical students facing food insecurity to inform sustainable, evidence-based solutions. Ultimately, addressing food insecurity within medical education is essential not only for improving student health and academic success but also for promoting equity and wellness across the future physician workforce.

## Appendices

Appendix A: Survey administered

The following questions were asked in the survey:

1. Age

2. Race/Ethnicity (American Indian or Alaska Native; Asian; Black or African American; Hispanic, Latino, or of Spanish origin; Native Hawaiian or Other Pacific Islander; White; and Other)

3. Academic year (3rd year, 4th year)

4. School (Institution A, Institution B)

5. Residency status (in-state, out of state)

6. Main clinical campus location (Three hospitals within the Healthcare Network)

7. Are you a first-generation college student? (yes/no)

8. Are you a first-generation medical student? (yes/no)

9. Do you receive financial aid (including loans)? (yes/no)

10. Is your family located within 10 miles of your clinical campus? (yes/no)

11. Do you currently use any of the following resources? (SNAP, Healthy Food Centers, Food pantries, Bellevue Farmers Market Food Assistance)

12. Do you know how to access these resources? (yes/no)
